# Antibacterial Activities of Lipopeptide (C_10_)_2_-KKKK-NH_2_ Applied Alone and in Combination with Lens Liquids to Fight Biofilms Formed on Polystyrene Surfaces and Contact Lenses

**DOI:** 10.3390/ijms20020393

**Published:** 2019-01-17

**Authors:** Malgorzata Anna Paduszynska, Magdalena Maciejewska, Katarzyna Ewa Greber, Wieslaw Sawicki, Wojciech Kamysz

**Affiliations:** 1 Department of Inorganic Chemistry, Faculty of Pharmacy, Medical University of Gdansk, 80-416 Gdansk, Poland; maciejewska.kj@gmail.com (M.M.); kamysz@gumed.edu.pl (W.K.); 2 Pharmaceutical Laboratory Avena Sp.z.o.o., 86-031 Osielsko, Poland; 3 Department of Physical Chemistry, Faculty of Pharmacy, Medical University of Gdansk, 80-416 Gdansk, Poland; greber@gumed.edu.pl (K.E.G.); wsawicki@gumed.edu.pl (W.S.)

**Keywords:** lipopeptides, biofilm, persister cells, ocular infections, biofilm on contact lenses

## Abstract

The widespread use of biomaterials such as contact lenses is associated with the development of biofilm-related infections which are very difficult to manage with standard therapies. The formation of bacterial biofilms on the surface of biomaterials is associated with increased antibiotic resistance. Owing to their promising antimicrobial potential, lipopeptides are being intensively investigated as novel antimicrobials. However, due to the relatively high toxicity exhibited by numerous compounds, a lot of attention is being paid to designing new lipopeptides with optimal biological activities. The principal aim of this study was to evaluate the potential ophthalmic application of lipopeptide (C_10_)_2_-KKKK-NH_2_. This lipopeptide was synthesized according to Fmoc chemistry using the solid-phase method. The antibiofilm activities of the lipopeptide, antibiotics used in ocular infections, and commercially available lens liquids were determined using the broth dilution method on polystyrene 96-well plates and contact lenses. Resazurin was applied as the cell-viability reagent. The effectiveness of the commercially available lens liquids supplemented with the lipopeptide was evaluated using the same method and materials. (C_10_)_2_-KKKK-NH_2_ exhibited stronger anti-biofilm properties compared to those of the tested conventional antimicrobials and showed the ability to enhance the activity of lens liquids at relatively low concentrations (4–32 mg/L). Estimation of the eye irritation potential of the lipopeptide using Toxtree software 2.6.13 suggests that the compound could be safely applied on the human eye. The results of performed experiments encourage further studies on (C_10_)_2_-KKKK-NH_2_ and its potential application in the prophylaxis of contact lens-related eye infections.

## 1. Introduction

Nowadays, the alarming growth and spread of antibiotic-resistant microorganisms is such a serious problem that it threatens the achievements of modern medicine [1]. Moreover, microorganisms form biofilms on the surface of biomaterials or human tissues that are up 1000 times more resistant to standard antibiotic therapy compared to their planktonic counterparts [2,3]. The continuing rise in antibiotic and multi-drug resistant as well as biofilm-related bacterial infections is a major global medical health issue and is associated with the failure of clinical treatment, the limitation of antibiotic use, and increased morbidity, mortality, and healthcare costs. All these factors significantly impact the world economy and, therefore, a new generation of antimicrobial compounds is required [4,5].

Cationic antimicrobial peptides (AMPs) are promising alternatives to conventional antibiotics, due to their unique mechanism of action that reduces the risk of bacteria developing resistance to them, and also because of their ability to inhibit multi-drug resistant bacterial biofilms [6,7]. AMPs, as part of the innate immune system, naturally occur in many parts of the human body. For instance, LL-37, defensins and psoriasin, as part of tear fluid, form an important part of the innate defense system in the human eye [8]. LL-37 is an intensively studied human AMP with confirmed anti-biofilm activity [9,10] that has provided a foundation on which to design numerous peptides to fight bacterial biofilm [11,12]. Natural AMPs, as well as their derivatives, have been investigated with regard to their potential ophthalmic use [13,14], including topical application [15,16], incorporation into contact lenses (CLs) [17,18], and as preservative agents in CL solution [14,19] and corneal storage media [20]. However, despite the promising results obtained with AMPs, their broad spectrum of antimicrobial activity and their low risk of resistance development, the application of these compounds in therapy is limited due to their potential toxicity, allergenicity, enzymatic degradation, poor stability in vivo, and high costs of production [21,22,23,24,25,26].

The research on features determining the antimicrobial activity of AMPs have yielded essential information for the design of novel, highly effective compounds with optimized biological properties that can also be produced at lower cost compared to their natural antimicrobial counterparts. Numerous studies focus on evaluating and designing shorter analogs, creating multimeric AMP-based sequences and developing peptidomimetics which can imitate the bactericidal mechanism of action.

A successful approach to modulating the activity and bioavailability of peptides is the acylation of cationic peptides with fatty acid. It has been shown that the introduction of d-amino acid or non-peptide residues significantly improves the antimicrobial spectrum activity of cationic peptides and determines a higher resistance to proteolytic degradation [27,28]. Simple modification, such as the acylation of short cationic residue, has resulted in short synthetic lipopeptides, a particularly promising group of compounds exhibiting a strong and broad spectrum of antimicrobial activity. They are composed of short positively-charged peptide chains conjugated with a fatty acid that provides amphipathicity. Those two features determine the surface-active properties of the compounds and allow them to electrostatically interact with a negatively-charged microbial membrane, leading to a rapid-kill drug-resistant pathogen [29]. The compounds are cost-effective and less time-consuming to produce in comparison with native AMPs.

So far, research has allowed numerous short lipopeptides endowed with high antibacterial as well as antifungal activity to be identified [21,30,31,32]. These lipopeptides have also been found to be effective against biofilms and multi-drug resistant bacteria [33]. However, their practical use in ophthalmology remains limited. Two critical issues are their potential toxicity or allergenicity [31,34]. These issues are especially important in the case of such a delicate and sensitive structure like the human eye and, therefore, a great deal of attention is being paid to optimizing the biological activities of lipopeptides.

In previous studies, we identified very a promising compound—(C_10_)_2_-KKKK-NH_2_—which exhibits strong antibacterial activities and low toxicity towards human cells in vitro [35,36]. In this study we have further investigated the antimicrobial activity of this compound with regard to its potential application in ophthalmology, and pre-evaluated its irritation potential via computational methods which have proved to be very useful in predicting and describing the properties of the compound [37,38,39].

## 2. Results

### 2.1. Activity of the Lipopeptide and Conventional Antibiotics against Biofilms Formed on Polystyrene

The tested compounds exhibited diverse antibiofilm activities towards various bacterial species. The durability of the antimicrobial effect after the withdrawal of the active compound varied significantly depending on the applied compound and the tested strain. In many cases, the removal of the antibiotic caused partial or even full renewal of bacterial biofilms.

**Figure 1:**
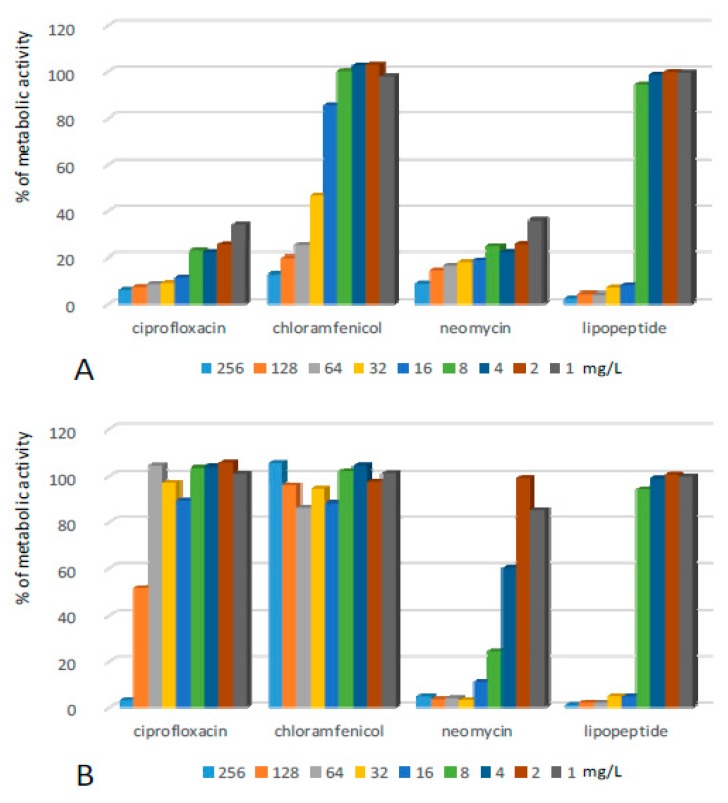
Activity of the lipopeptide and conventional antimicrobials applied at concentrations of 1–256 mg/L against SE biofilms formed on polystyrene (**A**) results read after 24 h exposure to compounds; and (**B**) results read after the withdrawal of compounds and an additional 24 h of incubation in MHB II. The results are presented as the percentage of metabolic activity in comparison to positive (100%) and negative (0%) controls; RSD ≤ 15%.

Structures formed by *Staphylococcus epidermidis* (SE) on the surface of 96-well plates turned out to be sensitive to all tested compounds (Figure 1, Table 1). The application of solutions of ciprofloxacin significantly reduced the metabolic activity of cells in the pre-grown structures. The antibiotic used at a range of concentrations from 1–8 mg/L caused a ca. 70–80% decrease in the metabolic activity of cultured bacteria, while concentrations of 16 mg/L and higher resulted in the reduction of metabolic activity to 10% and lower in comparison to the positive control. Additional incubation in the pure medium after the removal of solutions of ciprofloxacin (1–128 mg/L) caused an increase in the metabolic activity of the bacteria (Table 1). Only the concentration of 256 mg/L of the antibiotic created a permanent antibiofilm effect.

Similar antibiofilm activity against SE was presented by neomycin. However, in the case of this antibiotic, the effect remained after its withdrawal and additional incubation. Chloramphenicol exhibited rather low antibiofilm potential. In the first antibiofilm assay, the compound reduced the metabolic activity of bacteria to ca. 12, 20, and 25% once applied at concentrations of 256, 128, and 64 mg/L respectively. The activity was removed totally in the second assay—the metabolic activity of bacteria in all the samples after exposure and the subsequent withdrawal of chloramphenicol increased significantly. Lipopeptide (C_10_)_2_-KKKK-NH_2_ turned out to be very active against SE biofilm. Application at concentrations of 16–256 mg/L caused the metabolic activity of bacterial cells to reduce to ca. 5%. This was the strongest reduction of metabolic activity of SE cells observed in this assay. Moreover, the effect remained after additional incubation in the pure medium after the withdrawal of the lipopeptide.

**Figure 2:**
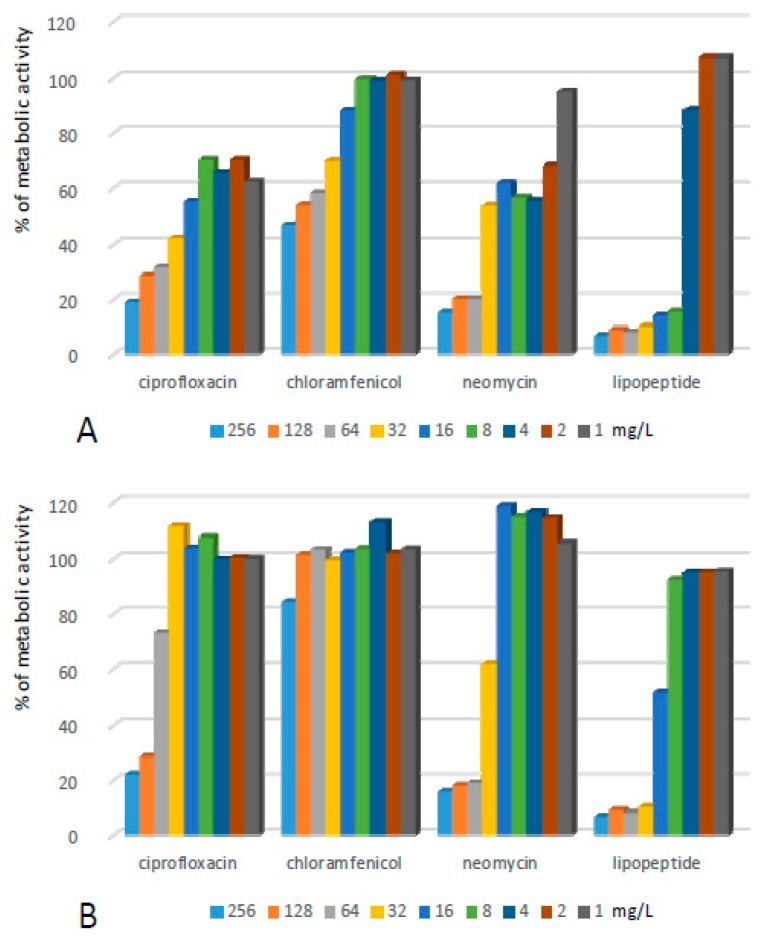
Activity of the lipopeptide and conventional antimicrobials applied at concentrations of 1–256 mg/L against SA biofilms formed on polystyrene (**A**) results read after 24 h exposure to compounds; and (**B**) results read after the withdrawal of compounds and an additional 24 h of incubation in MHB II. The results are presented as the percentage of metabolic activity in comparison to positive (100%) and negative (0%) controls; RSD ≤ 15%.

*Staphylococcus aureus* (SA) cultured on polystyrene plates turned out to be less sensitive in comparison to SE (Figure 2, Table 1). The difference in susceptibility is especially visible in the case of conventional antibiotics. Ciprofloxacin reduced the metabolic activity of SA to less than 20% when applied at the highest tested concentration and to ca. 30% when applied at concentrations of 128–164 mg/L. The antibiofilm effect was permanent only at the two highest concentrations (Table 1). The removal of the antibiotic SA in the sample treated with a concentration of 64 mg/L increased metabolic activity to ca. 70% of the positive control. Neomycin exhibited some higher and more permanent activity. However, the reduction of metabolic activity was not as significant as in the case of SE. The most potent antistaphylococcal agent was the lipopeptide. The compound caused a reduction in the metabolic activity of SA cells by over 90% when applied at concentrations of 32 mg/L and higher. The exposure to the lipopeptide caused a permanent antibiofilm effect—metabolic activity did not increase after the compound was replaced with pure MHB II. As in the case of SE, chloramphenicol was the least promising agent. The metabolic activity of SA was reduced by half only after the application of the compound at concentrations of 128–256 mg/L and increased significantly after the withdrawal of the antibiotic.

**Figure 3:**
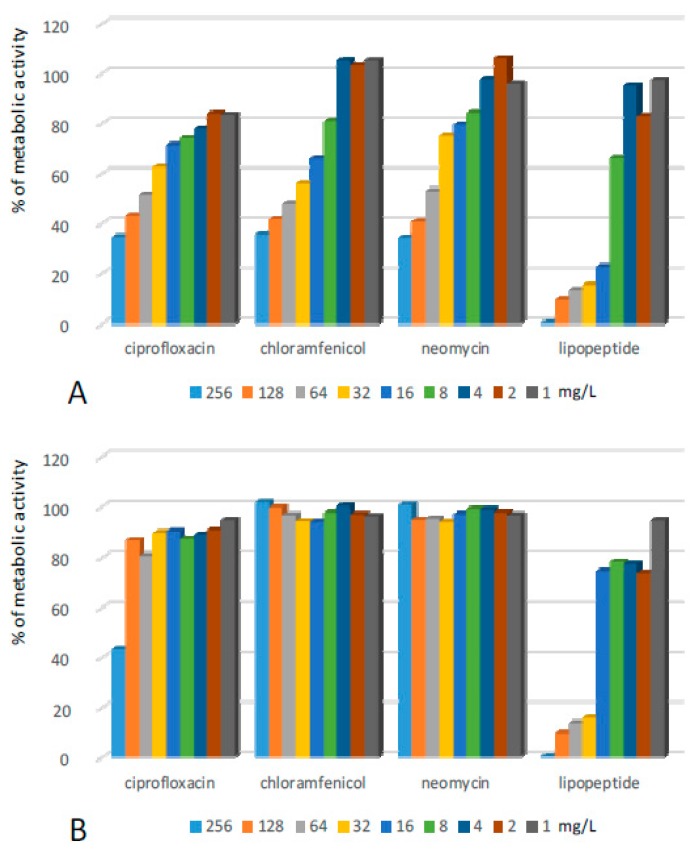
Activity of the lipopeptide and conventional antimicrobials applied at concentrations of 1–256 mg/L against EF biofilms formed on polystyrene (**A**) results read after 24 h exposure to compounds; and (**B**) results read after the withdrawal of compounds and an additional 24 h of incubation in MHB II. The results are presented as the percentage of metabolic activity in comparison to positive (100%) and negative (0%) controls; RSD ≤ 15%.

Biofilms formed by *Enterococcus feacalis* (EF) turned out to be the most resistant to conventional antimicrobials (Figure 3, Table 1). Application of all three compounds at the highest concentrations resulted in a reduction of metabolic activity in biofilms to ca. 35% of initial populations. After the withdrawal of antibiotics, the effect remained for ciprofloxacin, while the removal of chloramphenicol and neomycin resulted in the complete renewal of the metabolic activity of bacteria. The lipopeptide exhibited the ability to permanently eradicate the biofilm at concentrations of 32–256 mg/L. In both antibiofilm assays, a reduction of the metabolic activity of EF to less than 10% of the positive control was observed.

**Figure 4:**
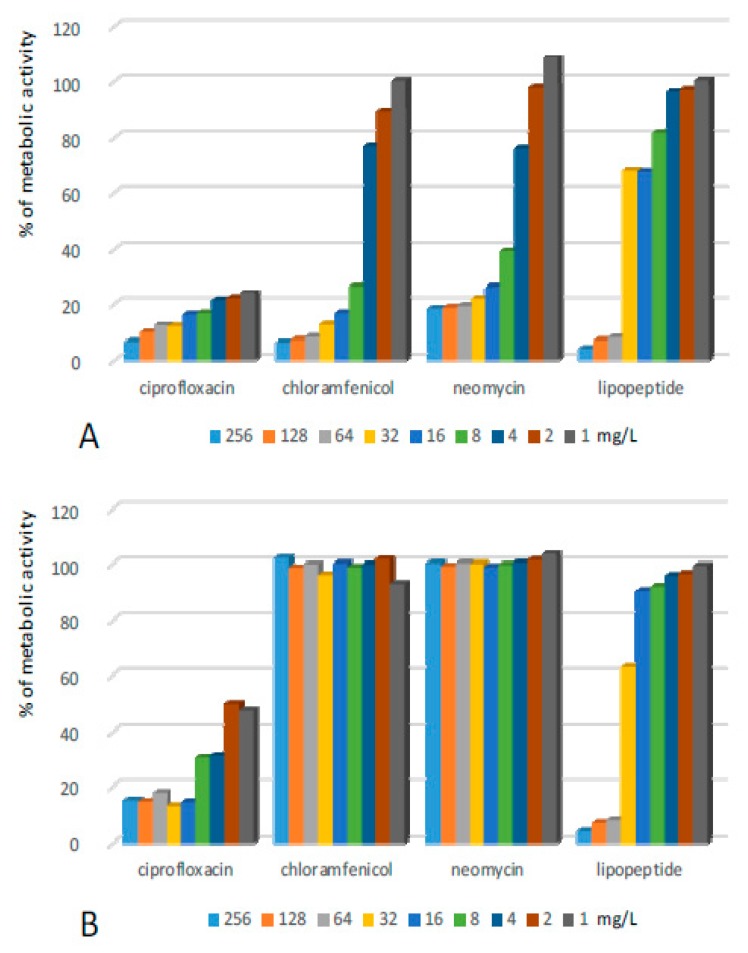
Activity of the lipopeptide and conventional antimicrobials applied at concentrations of 1–256 mg/L against EC biofilms formed on polystyrene (**A**) results read after 24 h exposure to compounds; and (**B**) results read after the withdrawal of compounds and additional 24 h of incubation in MHB II. The results are presented as the percentage of metabolic activity in comparison to positive (100%) and negative (0%) controls; RSD ≤ 15%.

Some higher concentrations of the lipopeptide were required to fight structures formed by *Escherichia coli* (EC) (Figure 4, Table 1). This effect was observed after application of the lipopeptide at concentrations of 64 mg/L and higher. However, the reduction of metabolism was also very high and did not deteriorate after the withdrawal of the compound. A similar effect was observed after the exposure of EC biofilms to ciprofloxacin at concentrations of 32–256 mg/L. Treatment with lower concentrations reduced metabolic activity by 75 to 85%, however, after removal of the antibiotic, a certain increase of metabolic activity was observed. Chloramphenicol, as well as neomycin, also exhibited rather high effectiveness towards EC biofilms; however, the bacterial populations were able to fully restore their metabolic activity when the compounds were removed from the environment.

**Figure 5:**
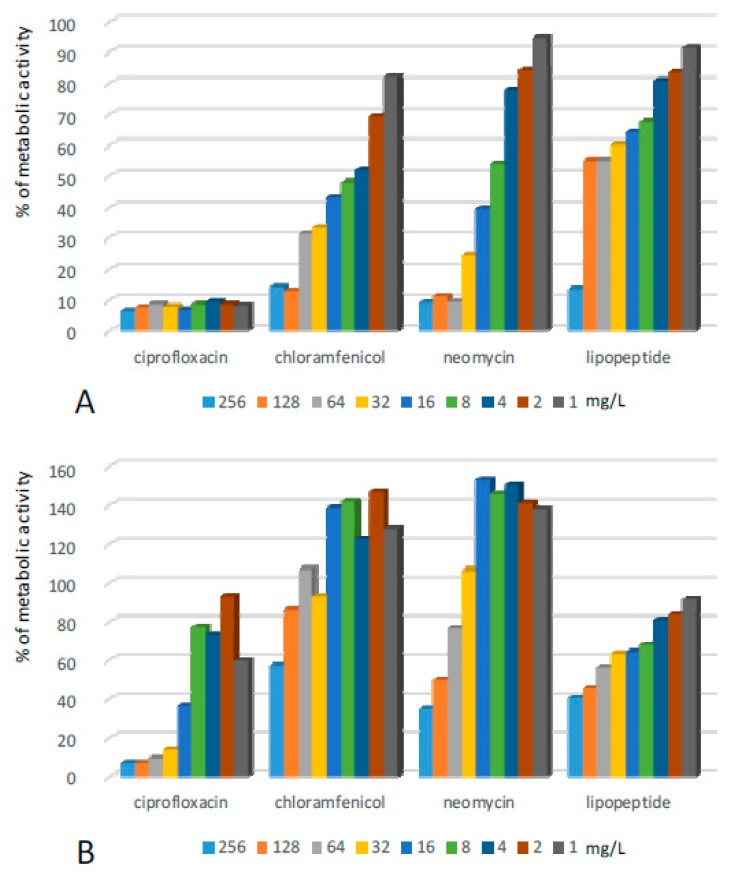
Activity of the lipopeptide and conventional antimicrobials applied at concentrations of 1–256 mg/L against PA biofilms formed on polystyrene (**A**) results read after 24 h exposure to compounds; and (**B**) results read after the withdrawal of compounds and additional 24 h of incubation in MHB II). The results are presented as the percentage of metabolic activity in comparison to positive (100%) and negative (0%) controls; RSD ≤ 15%.

*Pseudomonas aeruginosa* (PA) formed a biofilm which exhibited the highest resistance towards the lipopeptide (Figure 5, Table 1). The compound reduced the metabolic activity of bacteria to 10% only when applied at a concentration of 256 mg/L. Unfortunately, the bacteria repopulated and gained 40% of the metabolic activity of the positive control after the removal of the lipopeptide. Application of lower concentrations resulted in a 50% decrease of bacterial metabolism which did not increase after the withdrawal of the compound. Ciprofloxacin was highly active against PA—it reduced the metabolic activity of bacteria by more than 90% even at the lowest applied concentrations. Further incubation in the medium without antibiotics caused a certain renewal of metabolic activity within the biofilm, but only in wells treated with the antibiotic applied at concentrations lower than 32 mg/L. Exposure of the PA biofilm to chloramphenicol at concentrations of 128–256 and 32–64 mg/L resulted in the reduction of the metabolic activities of bacteria by ca. 90% and 70%, respectively. The incubation of PA after replacing solutions of chloramphenicol with MHB II resulted in a significant increase in metabolism. Interestingly, pretreating the biofilm with the compound at concentrations lower than 32 mg/L resulted in a significant promotion of biofilm growth. Very similar results were obtained for neomycin. The compound reduced metabolic activity by over 90% at concentrations of 64–256 mg/L and 75% after the exposure of PA to the compound at a concentration of 32 mg/L. Removal of the antibiotic resulted in a significant increase of PA metabolism. As in the case of chloramphenicol, pretreatment of a PA biofilm with concentrations lower than 32 mg/L caused the enhanced metabolism of bacteria in comparison to the positive control.

### 2.2. Activity of Lipopeptide and CL Solutions against Biofilms Formed on CLs

**Figure 6:**
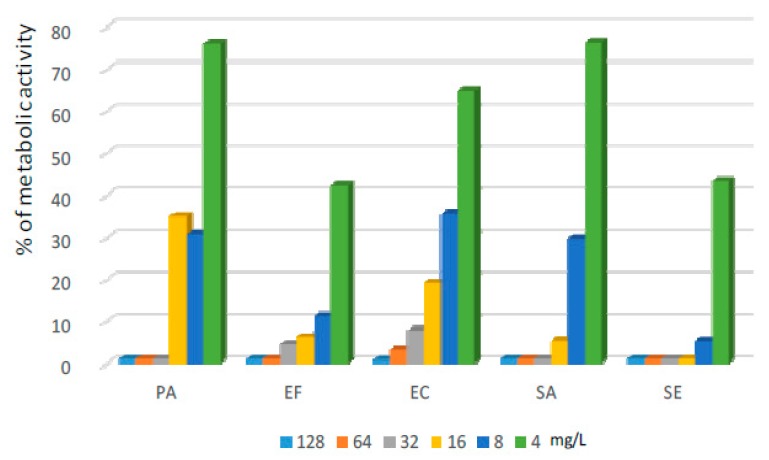
Activity of the lipopeptide applied at concentrations 4–128 mg/L against biofilms formed on CLs. The results are presented as the percentage of metabolic activity in comparison to positive (100%) and negative (0%) controls; RSD ≤ 20%.

Commercially-available lens liquids proved to be highly active against biofilms formed on CLs. They reduced the metabolism of bacteria cultured on CLs by at least 90% for the vast majority of tested strains. Both liquids A and B caused a reduction of bacterial metabolism to 10% of the positive control (or lower) for SA, SE, EF, and EC, while only liquid A demonstrated this activity against PA. The application of the lipopeptide dissolved in PBS allowed biofilms formed by all tested strains to be removed from the CLs. The highest effectiveness was observed for SE and EF. After exposure to the lipopeptide at a concentration of 8 mg/L, the metabolic activity of bacteria reduced by at least 90%. For such a significant decrease of metabolism of SA cells, the application of the lipopeptide at a concentration of 16 mg/L was needed. The most difficult cultures to eliminate with lipopeptide solutions were EC and PA—to reduce the metabolic activity of these strains by more than 90%, a concentration of 32 mg/L of lipopeptide were required (Figure 6).

These very promising results were obtained when the exposure to CL liquids and solutions of lipopeptide lasted until the reading of results. Once the CL liquids were removed and the CLs were further incubated in MHB II, the metabolic activities of bacterial populations of the majority of strains were nearly fully renewed. The antibacterial effect of CL solutions remained only for SE, while for liquid A and the EF strain, biofilm growth increased. Some better, but also not fully satisfying, results were obtained for the lipopeptide. It permanently removed SA and SE biofilms from CLs once applied at a concentration of 16 mg/L. For Gram-negative strains, the bacterial populations regrew to a high extent (PA was fully restored, EC to ca. 50%) after the removal of the lipopeptide at all tested concentrations. Similarly as in the case of CL solution A, treating EF with the lipopeptide promoted bacterial growth.

### 2.3. Antibiofilm Activity of the Lipopeptide Applied in Combination with CL Liquids

#### 2.3.1. Biofilms Formed on Polystyrene Surfaces

**Figure 7:**
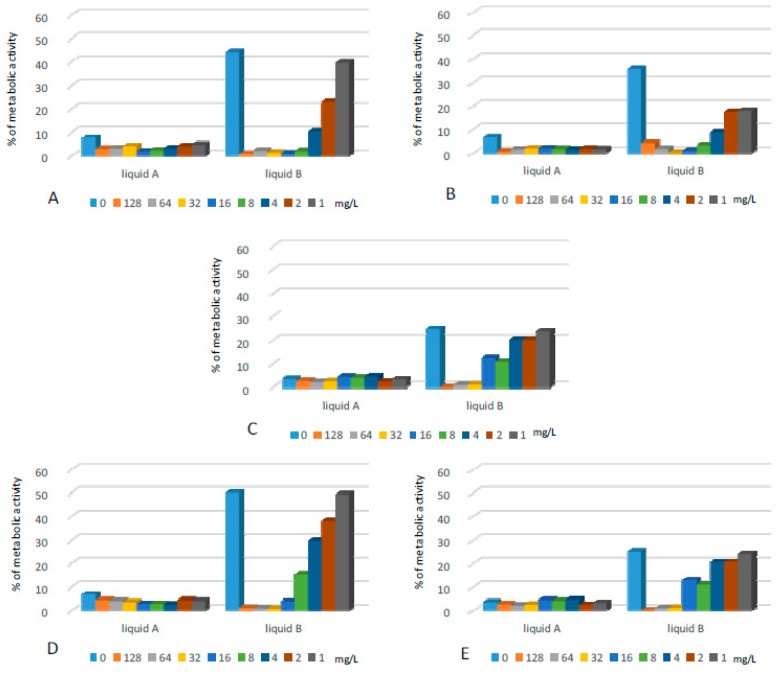
Activity of CL liquids alone and supplemented with the lipopeptide applied at concentrations of 1–128 mg/L against biofilms formed on polystyrene plates by: (**A**) SE; (**B**) SA; (**C**) EF; (**D**) EC; and (**E**) PA. The results are presented as the percentage of metabolic activity in comparison to positive (100%) and negative (0%) controls; RSD ≤ 15%.

In this assay we found that the supplementation of CL solutions with lipopeptide positively influenced their antibacterial activity (Figure 7). This was especially noticeable for liquid B, which demonstrated lower effectiveness in comparison to liquid A. The latter caused the reduction of metabolic activity of bacteria by more than 90% for almost all tested strains, but supplementation with the lipopeptide resulted in an even higher decrease of microbial metabolism. Exposure of PA biofilms to liquid A reduced biofilm metabolism to ca. 15% of the positive control, while supplementation with the lipopeptide at a very low concentration (1 mg/L) caused further reduction in bacterial metabolic activity - to 5% of positive control.

Liquid B applied alone caused only partial reduction of bacterial metabolic activity for the majority of strains. Its supplementation with the lipopeptide at a concentration of 4–8 mg/L reduced the biofilms of SE, SA, EC, and EF by at least 90%. The PA biofilm was not affected by liquid B at all. Its combination with the lipopeptide at concentrations of 128 and 64 mg/L reduced the bacterial metabolism to ca. 4 and 20%, respectively.

The lipopeptide applied alone was highly active against biofilms formed by SA and SE at concentrations of 32 and 16 mg/L, respectively. Application of the same concentration of the lipopeptide reduced metabolic activity in the EF biofilm by ca. 95%. Gram-negative strains cultured on polystyrene surfaces turned out to be much less sensitive to the lipopeptide, which caused partial reduction of bacterial metabolism. For PA, we observed medium antibacterial activity only at the highest concentration—128 mg/L.

#### 2.3.2. Biofilms Formed on CLs

**Figure 8:**
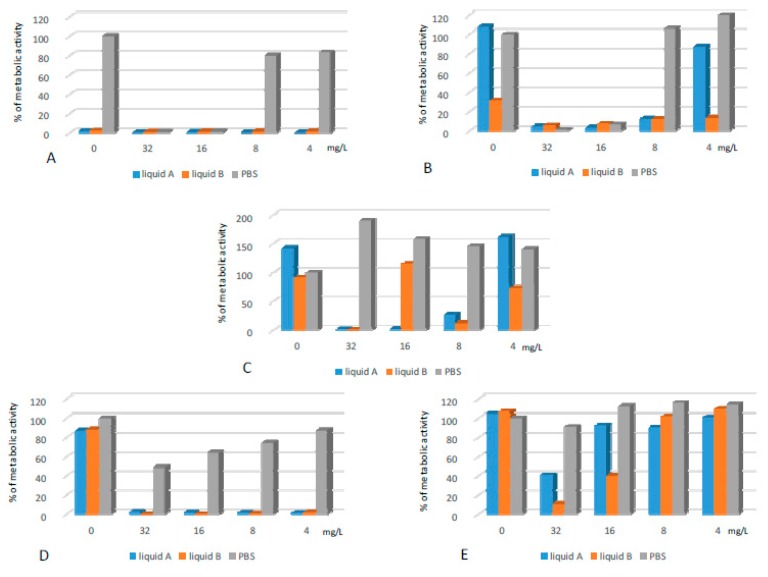
Activity of CL liquids alone and supplemented with the lipopeptide applied at concentrations of 4–32 mg/L against biofilms formed on CL by: (**A**) SE; (**B**) SA; (**C**) EF; (**D**) EC; and (**E**) PA. The results are presented as the percentage of metabolic activity in comparison to positive (100%) and negative (0%) controls; RSD ≤ 20%.

As described in Section 2.2, the antibacterial effect of CL solutions as well as the lipopeptide applied alone does not last once the agents are replaced with MHB II. Supplementation of CL solutions with the lipopeptide significantly improved their antibiofilm activity, which remained after the withdrawal of active agents (Figure 8). The synergistic effect was especially noticeable for PA, EC and EF. When the CL solutions or lipopeptide were applied alone against EC and PA biofilms, the metabolic activities of bacteria were partially or fully restored. Supplementation of both liquids with the lipopeptide at a concentration of 4 mg/L reduced bacterial metabolism to ca. 2%. A permanent and sufficient activity against PA was obtained after the application of liquid B with the lipopeptide at a concentration of 32 mg/L. To nearly totally remove (metabolic activities ca. 1–2%) the EF biofilms from the surface of CLs, liquids A and B were supplemented with the lipopeptide at concentrations of 16 and 32 mg/L, respectively. SA was permanently eliminated from CLs once the lipopeptide was added at concentrations of 8 and 4 mg/L to liquid A and B, respectively. Despite the fact that SE biofilms were susceptible to the agents when applied alone, we also noticed a synergistic effect between the lipopeptide and CL solutions.

### 2.4. Eye Corrosion

Obtained result suggested that target structures do not show eye irritation properties as well as do not cause skin corrosion.

## 3. Discussion

The handling of CLs after insufficient hand washing, CL storage cases and solutions may be potential sources of contamination causing the development of CL-associated infections, which are relatively rare, but can pose severe vision-threatening complications [40,41,42,43]. Due to the development of bacterial resistance as well as the existence of bacteria in the form of biofilms, the standard means of prevention and treatment of ocular infections are not always sufficient. Numerous AMPs and their derivatives have been investigated as potential alternatives to conventional antibiotics, as well as disinfecting solutions.

The topical application of a cecropin-melittin hybrid was effective in a pseudomonas keratitis model in rabbits [16]. Another hybrid (protamine-melittin) peptide—melimine—was successfully evaluated as an antimicrobial CL coating for the prevention of contact lens-induced acute red eye (CLARE) in the PA guinea pig model [17] and contact lens-induced peripheral ulcer (CLPU) in the rabbit model [18]. CLs coated with the peptide were also tested in a human clinical trial, where they demonstrated broad spectrum, high antimicrobial activity and turned out to be safe for use [44]. A derivative of melimine—mel4—was recently successfully applied as an antimicrobial coating for silicone hydrogel CLs [45].

Peptide Shiva-11—a synthetic analogue of cecropin—was found to be effective against PA, SE, and SA as an antibacterial agent in CL solutions [46]. In another study, the peptide demonstrated a wide range of antimicrobial activity against pathogens isolated from a human suffering from ocular infections [14]. Another analogue of cecropin—D5C—exhibited the ability to enhance the effectiveness of commercially-available disinfecting solutions [19]. The lipopeptide used in our study also demonstrated the potential to enhance the antimicrobial activity of commercial CL solutions.

For our study, we chose the CL liquids which were the most effective in our previous work [47]. As in the previous work, the application of liquid A eliminated at least 90% of living cells of all strains cultured on CL. Liquid B was effective against the majority of strains except PA. When applied alone, the lipopeptide demonstrated high activity against Gram-positive bacteria and some lower activity against Gram-negative strains.

A further assay revealed that the above activities do not last if the CL solutions, and in some cases also the solutions of lipopeptide, are removed from the environment. This suggests that a certain population of microbial cells survived the exposure to tested solutions and repopulated after the removal of these solutions. Further evaluation should be performed in order to determine if the remaining cells can be identified as persister cells. Persister cells are considered to be responsible for the resistance of biofilm to antimicrobial agents. They constitute a small population of microbial cells which exist in the presence of antibiotics, have low metabolic activity and do not grow. They are believed to be responsible for the return of infections after the withdrawal of antibiotic treatment [48]. It has not been determined if the repopulation of biofilms in the study was the result of the presence of persisters or regular cells protected by exopolysaccharide (EPS) or other resistance mechanisms. Therefore, the results are interpreted as permanent/non-permanent antimicrobial activity.

The obtained results revealed that the antimicrobial effect of CL solutions was permanent only in the case of SE, while the lipopeptide turned out to be active only against staphylococci. Interestingly, exposure of EF to the lipopeptide resulted in the promotion of biofilm growth on CLs after the solution was replaced with MHB II. This may be explained by the defense mechanism of bacteria. Biofilm formation is described as a mechanism developed by bacteria in order to avoid the action of human AMPs in a human body [49,50,51]. In order to assess if there is synergistic activity between CL solutions and the lipopeptide, bacterial biofilms cultured on polystyrene were exposed to CL solutions supplemented with the compound. We observed a positive influence of lipopeptide supplementation on the effectiveness of applied CL solutions against all tested strains, and due to this, an assay with CLs was performed. As expected, the supplementation of CL liquids with the lipopeptide achieved permanent disinfection of CLs. For staphylococci and EC, this was observed after usage of the lipopeptide at a concentration of 4 mg/L, while for EF some higher concentrations were necessary. For PA, only usage of liquid B with the lipopeptide at 32 mg/L gave satisfying results.

PA was also the most difficult strain to eliminate from the surface of CL in the previous study, in which we investigated amphibian peptides and short lipopeptides containing hexadecanoic acid according to their potential application as CL solution additives [47]. The results of performed antimicrobial assays were also very promising. However, due to high toxicity towards human keratinocytes in vitro, the ocular applications of lipopeptides with hexadecanoic acid are not worth further consideration [31,36]. In contrast to those lipopeptides, the compound with two residues of decanoic acid does not exhibit toxicity towards human cells in vitro at its microbiologically-active concentrations [35,36]. The results obtained in the present study revealed that the lipopeptide enhances the activity of commercial CL solutions at concentrations much lower than the ones identified as toxic to both human keratinocytes and erythrocytes. Moreover, according to the results obtained by the computational method, the compound is expected not to irritate the human eye. This needs to be confirmed with experimental methods, but data collected so far suggest that (C_10_)_2_-KKKK-NH_2_ might be a promising antibacterial additive to CL solutions.

The results obtained in assays on polystyrene plates demonstrated that the compound could also be worth further consideration as an alternative to antibiotic therapy of biofilm-related infections. In previous studies we confirmed the high antibiofilm activities of lipopeptides containing hexadecanoic acid [21,52]. However, due to high toxicity in vitro, the compounds should not be further considered for administration other than topical skin application [31,36]. (C_10_)_2_-KKKK-NH_2_ demonstrates the ability to eradicate a bacterial biofilm at slightly higher concentrations in comparison to lipopeptides with hexadecanoic acid. However, for the majority of strains, the active concentrations were identified as safe to human cells. Moreover, the results obtained for the lipopeptide in the case of Gram-positive bacteria are much more satisfying in comparison to those obtained for conventional antimicrobials. The compound demonstrated the ability to permanently eliminate the living bacterial cells of EF, SA, and SE once applied at concentrations of 32 (EF, SA) and 16 (SE) mg/L, which are below the concentrations identified as toxic to human cells. For SE, similar results were obtained for neomycin: the antibiotic was also active at a concentration of 16 mg/L. However, the reduction of bacterial metabolism was not as significant as was demonstrated by the lipopeptide. The antibiotic was also active against SA, but at some higher concentration in comparison to the lipopeptide. The remaining conventional antimicrobials exhibited certain activity in the first assay, however after the withdrawal of compounds, the bacteria repopulated to a high extent. EF cultured on polystyrene plates turned out to be not sensitive to the action of all conventional antibiotics. The number of living cells reduced somewhat at higher concentrations (64–256 mg/L), but after the removal of antibiotics, the bacteria repopulated almost completely. Based on these results, we can expect the clinical failure of application of these compounds for biofilm-related infections. Even in the case of a positive effect of therapy, a return of infection can be expected after the treatment is completed.

Difficulties in the elimination of SA and EF biofilms with ciprofloxacin have previously been reported [53]. Ciprofloxacin exhibited much higher activity against Gram-negative bacteria, which was to be expected as the infections caused by EC are the main therapeutic indications for the application of this antimicrobial. The structures were permanently removed from polystyrene after application of the compound at a concentration of 16 mg/L, while to eradicate PA a concentration of 32 mg/L was sufficient. The lipopeptide demonstrated the activity at a concentration of 64 mg/L in the case of EC, but full elimination of PA was not achieved at the tested range of concentrations: even after application of the peptide at 256 mg/L, the bacterial population regrew significantly (to 40%). Chloramphenicol and neomycin showed rather weak activity against EC and PA biofilms, moreover the bacteria repopulated to a high extent after the withdrawal of compounds, even after exposure to their highest concentrations. The obtained results suggest that for biofilm-associated infections, ciprofloxacin can be recommended for Gram-negative infections and neomycin is expected to be effective against staphylococcal infections, while chloramphenicol is ineffective in the fight against biofilms of all tested strains. The lipopeptide shows high activity against biofilms formed by Gram-positive bacteria and is definitely worth further testing in this regard. Its concentrations that were active against biofilms are only a few times higher in comparison to the previously determined minimum inhibitory concentrations [35], while for conventional antimicrobials, concentrations at least 50–100 times higher in comparison to MICs are needed to eradicate biofilms [21,52].

According to the literature, the mechanisms responsible for biofilm resistance/persistence include the protection of microbial cells by the presence of EPS, changes in gene expression, the slowing down of metabolism and the presence of persister cells [54,55]. Promising results obtained for lipopeptides can be explained by their mechanism of action, based on interactions with microbial cell membranes, which allows slow- or even non-growing bacteria to act. Moreover, the small size of the molecules as well as their surfactant activity probably facilitates their penetration through EPS.

The recalcitrance to eradication by antibiotics is described as a characteristic feature of the bacteria in biofilms. Even after exposure to high doses of an antimicrobial, a fraction of cells can survive and repopulate once the antibiotic is withdrawn, leading to secondary infection [56]. According to the obtained results, this is not to be expected after treatment with the lipopeptide as the compound seems to eliminate all the bacteria within the biofilm. It was previously reported that small molecules and AMPs demonstrate the ability to kill persister cells [57].

The excellent antimicrobial activity was previously reported for many lipopeptides. This, along with the relatively low production costs, has encouraged many research groups to study their antimicrobial activities and design molecules with optimal properties. C_16_-KK-NH_2_ is one of such extensively studied compounds. As mentioned, the lipopeptide exhibits broad spectrum, high antimicrobial activity, including antibiotic-resistant strains as well as biofilm-associated bacteria [21,58], but also demonstrates high toxicity towards human cells at very low concentrations [36]. Other derivatives of hexadecanoic acid containing short-sequence peptides with alanine, glycine, leucine and lysine have demonstrated strong activity against SA strains [59]. Lipopeptides containing tryptophan and ornithine residues combined with capric, caproic, caprylic, lauric, myristic, and palmitic acids, and combinations of lauric acid with short sequences composed of ornithine and cysteine exhibited similar activities [21,60].

There is no doubt that lipopeptides are a very interesting alternative for the therapy of biofilm-related or drug-resistant microbial infections. The main limitation is their high toxicity resulting from their non-specific mechanism of action. The compounds disrupt the membranes of red blood cells when the cells are exposed to the compounds at concentrations close to their minimum inhibitory concentrations [61,62]. Therefore, the need to design new molecules with optimal properties is very urgent. (C_10_)_2_-KKKK-NH_2_ is an example of a successfully designed and synthesized novel compound based on AMPs.

## 4. Materials and Methods

### 4.1. Bacterial Strains and Culture Conditions

Bacterial strains were obtained from the Polish Collection of Microorganisms (Polish Academy of Science, Wroclaw, Poland). Three Gram-positive and two Gram-negative strains linked with CL-related infections were chosen for the study (*Staphylococus aureus* ATCC 6538, *Staphylococcus epidermidis* ATCC 14990, *Pseudomonas aeruginosa* ATCC 9027, *Escherichia coli* ATCC 25922, and *Enterococcus faecalis* ATCC 29212). The bacteria were cultured in a Mueller Hinton Broth II (MHB, Biocorp, Warsaw, Poland) overnight, under aerobic conditions at 37 °C. After incubation, the liquid cultures were centrifuged (2500 rpm for 10 min) and washed with phosphoric buffer (PBS, AppliChem, Darmstadt, Germany) three times and resuspended in fresh MHB II for inoculums appropriate for the performed assays.

### 4.2. Antimicrobials and CL Liquids

Ciprofloxacin, chloramphenicol and neomycin (sulfate) were purchased from Sigma-Aldrich, (St. Louis, MO, USA). Lipopeptide (C_10_)_2_-KKKK-NH_2_ was synthesized in the Department of Physical Chemistry (Medical University of Gdansk, Gdansk, Poland) according to the previously described protocol [62]. Two commercially-available popular CL solutions with the following compositions were tested:

A: Citrate, Tetronic 1304, aminomethylpropanol, sodium chloride, boric acid, sorbitol, disodium edetate, Polyquad (Polyquaternium) 0.001%, Aldox (myristamidopropyl dimethylamine) 0.0005%.

B: Boric Acid, disodium edetate, sodium borate, sodium chloride, DYMED (polyaminopropyl biguanide) 0.0001%, HYDRANATE (hydroxyalkylphosphonate) 0.03%, Poloxamine 1%.

### 4.3. Activity of Lipopeptide and Antibiotics against Biofilms Formed on 96-Well Plates

Bacterial suspensions were added to 96-well plates (Kartell, Noviglio, Italy) at initial inoculums of ca. 5 × 10^8^ CFU/mL and incubated under aerobic conditions with shaking (120 rpm) at 37 °C for 24 h. After this time, the wells were washed three times with PBS, and fresh medium supplemented with the lipopeptide and antibiotics was added. The bacterial cultures were exposed to graded concentrations (range 1–256 mg/mL) of antimicrobials in MHB II for 24 h (aerobic conditions, 120 rpm shaking, 37 °C). After exposure, the wells were washed three times with PBS and a solution of resazurin (Sigma Aldrich, St. Louis, MO, USA) in MHB II (0.01%) was added. This cell viability reagent is metabolized by bacterial dehydrogenases upon contact with living cells. As a result, the blue dye is reduced to a pink resorufin. After 1.5 h of incubation, the absorbance was measured at 570 and 600 nm using a microplate reader (Thermo Fisher Scientific, Waltham, MA, USA). The results are presented as a % of living cells (metabolic activity) compared to the positive control (sample with bacteria suspended in pure MHBII) and negative control (pure MHB II), which were taken as 100% and 0%, respectively. The metabolic activity of bacteria in the samples was measured according to the following formula:

Metabolic activity (%) = (ΔAbs of sample − ΔAbs of negative control)/(ΔAbs of positive control − ΔAbs of negative control);

ΔAbs = absorbance at 570 nm − absorbance at 600 nm;

The presented results are the means of nine results obtained on three different days.

### 4.4. Activity of Lipopeptide and Antibiotics against Biofilms Formed on 96-Well Plates after the Withdrawal of the Applied Antimicrobial

This assay was a continuation of assay 4.3 and was performed in order to assess the durability of the antibiofilm effect. The procedure was conducted as described in Section 4.3, with the difference that after exposure to antimicrobials, the wells were washed three times with PBS and pure MHB II was added. The samples were incubated for another 24 h (aerobic conditions, 120 rpm shaking, 37 °C). Then the medium was replaced with resazurin in MHB II (0.01%). The absorbance was measured at the same wave lengths and metabolic activity was calculated according to the formula given in Section 4.3. The presented results are means of nine results obtained on three different days.

### 4.5. Activity of Lipopeptide and CL Liquids against Biofilms Formed on CLs

Bacterial biofilms were cultured on commercially available CLs (1-Day Acuvue Moist, containing Etafilcon A, obtained from Johnson and Johnson Vision Care, Jacksonsville, FL, USA). The CLs were placed in polystyrene 24-well plates (Orange Scientific, Braine-l’Alleud, Belgium) in bacterial suspensions in MHB II at initial inoculums of ca. 5 × 108 CFU/mL. After 24 h of incubation (aerobic conditions, 120 rpm shaking, 37 °C), all of the CLs were rinsed three times with PBS. The lenses were then transferred into new wells with CL liquids and solutions of the lipopeptide in PBS at graded concentrations (range 4–64 mg/mL) and incubated again for 24 h at 37 °C. After incubation, resazurin was added (final concentration per sample = 0.01%) and absorbance was measured as in the assays 4.3 and 4.4. Positive controls contained CLs with bacterial biofilms placed in pure PBS, while sterile CLs incubated in MHB II replaced with PBS served as negative controls. The experiments were performed in triplicate on three different days.

### 4.6. Antibiofilm Activity of the Lipopeptide Applied in Combination with Commercially-Available Lens Liquids

#### 4.6.1. The Effect of the Lipopeptide on the Effectiveness of the Lens Liquids against Biofilms Formed on 96-Well Polystyrene Plates

The bacteria were cultured as described in Section 4.3 and afterwards exposed to graded concentrations (range 128mg/mL) of the lipopeptide dissolved in PBS and the CL solutions. The assay was also performed for the samples where biofilms were exposed to CL liquids without the lipopeptide. Positive controls (100%) were wells with pre-grown biofilms where the MHB II was replaced with pure PBS, while negative controls were wells with pure MHB II (also replaced with PBS). After 24 h of incubation (aerobic conditions, 120 rpm shaking, 37 °C), the solutions were replaced with resazurin in MHB II and the results were read and presented as described in Section 4.3.

#### 4.6.2. Activity of the Lipopeptide, Lens Liquids and Their Combinations against Biofilms Formed on CLs after Withdrawal of the Antimicrobial Solution

Biofilms on CLs were grown as described in Section 4.5 and exposed to graded concentrations of the lipopeptide dissolved in CL solutions and PBS. The assay was also performed for the samples where biofilms on CLs were exposed to pure CL solutions. Positive controls (100%) were biofilms on CLs in PBS, while negative controls were CLs previously incubated in pure MHB II replaced for the exposure time with PBS. After 24 h of exposure (aerobic conditions, 120 rpm shaking, 37 °C) all the solutions were removed, replaced with MHB II and the samples were incubated for another 24 h. Resazurin was then added in order to visualize the results. The results were read, calculated, and presented as in the previously-described sections.

### 4.7. Eye Irritation Calculation Assay

The eye irritation of investigated structures was calculated using Toxtree software 2.6.13 (free and available on the web site http://toxtree.sourceforge.net/) based on the “Estimates eye irritation and corrosion potential by physicochemical property ranges and structural rules“ algorithm implemented into a decision tree [63].

## Figures and Tables

**Table 1:** Activities of conventional antimicrobials and the lipopeptide—(C_10_)_2_-KKKK-NH_2_ against bacterial biofilms formed on polystyrene plates presented as MBEC—minimum biofilm eradication concentration (mg/L); MBEC 90—the lowest concentration which allowed to reduce the metabolic activity of bacteria by at least 90 ± 5%; MBEC 90 II – the lowest concentration which resulted in permanent reduction of metabolic activity by at last 90 ± 5%; MBEC 50—the lowest concentration which allowed to reduce the metabolic activity by at least 50 ± 5%; MBEC 50 II—the lowest concentration resulted in permanent reduction of the metabolic activity by at least 50 ± 5%.

Compound	MBEC 90	MBEC II 90	MBEC 50	MBEC II 50
	***Staphylococcus epidermidis***			
Ciprofloxacin	16	256	≤1	128
Chloramphenicol	256	>256	32	>256
Neomycin	16	16	≤1	8
Lipopeptide	16	16	16	16
	***Staphylococcus aureus***			
Ciprofloxacin	>256	>256	16	128
Chloramphenicol	>256	>256	128	>256
Neomycin	64	64	4	64
Lipopeptide	32	32	8	16
	***Enterococcus feacalis***			
Ciprofloxacin	>256	>256	64	256
Chloramphenicol	>256	>256	32	>256
Neomycin	>256	>256	64	>256
Lipopeptide	32	32	16	32
	***Escherichia coli***			
Ciprofloxacin	32	32	≤1	≤1
Chloramphenicol	16	>256	8	>256
Neomycin	>256	>256	8	>256
Lipopeptide	64	64	64	64
	***Pseudomonas aeruginosa***			
Ciprofloxacin	≤1	32	≤1	16
Chloramphenicol	128	>256	4	256
Neomycin	64	>256	8	128
Lipopeptide	256	>256	64	64

## Abbreviations

AMP	Antimicrobial peptide
CL	Contact lens
EC	*Escherichia coli*
EF	*Enterococcus faecalis*
MHB II	Mueller Hinton Broth II
SA	*Staphylococcus aureus*
SE	*Staphylococcus epidermidis*
PA	*Pseudomonas aeruginosa*
PBS	Phosphoric buffer
